# Atypical Takotsubo Post-Natural Delivery: A Guideline-Based Approach for Diagnosing Takotsubo Cardiomyopathy

**DOI:** 10.7759/cureus.74580

**Published:** 2024-11-27

**Authors:** Abdelrahman Kenawi, Neeraj Joshi, Ahmed Mohamed

**Affiliations:** 1 Cardiology, East Kent Hospitals University NHS Foundation Trust, Margate, GBR; 2 Cardiology, Eastbourne District General Hospital, East Sussex Healthcare NHS Trust, Eastbourne, GBR

**Keywords:** atypical takotsubo cardiomyopathy, natural delivery, post partum, stress-related cardiomyopathy, tako-tsubo cardiomyopathy (ttc)

## Abstract

Since its first description in 1990, Takotsubo cardiomyopathy (TC) has continued to puzzle physicians and scientists alike as the mechanisms behind the characteristic unique left ventricular dysfunction that marks the condition and its relation to the intense emotional and physiological stressors that usually precede it have remained not fully understood. Since then, several different variants of the condition have been described that do not conform to the conventional narrative of a post-menopausal disease that only affects women of a certain age and is triggered by a preceding event. Of these variants, atypical Takotsubo, which does not assume the typical characteristic apical ballooning that marks the condition has been linked with a younger age group and a more neurological trigger. A strong guideline-based clinical suspicion and an astute knowledge of its clinical, laboratory, and different morphological presentations are often needed to differentiate the case from acute coronary syndromes, which it so closely resembles. This case illustrates an atypical presentation of atypical TC in an otherwise healthy woman and how a guideline-based approach can be utilized to identify and manage the condition while discussing the most recent theories addressing TC.

## Introduction

First described in 1990 by Sato et al. [1], Takotsubo cardiomyopathy (TC) is characterized by acute left ventricular dysfunction triggered by intense physical or emotional stressors. This makes it challenging to differentiate from acute myocardial infarction (MI), with approximately 0.75% to 2.5% of all initially suspected acute coronary syndrome (ACS) cases ultimately diagnosed as TC [2]. The condition takes its name from the contraption used to catch octopuses, as the acutely dysfunctional left ventricle assumes a similar morphological appearance during systole. This is, by far, the most common type of TC and is characterized by apical ballooning and basal hyperkinesia. Since its first description, other phenotypes have been described including (focal, reversed, and mid-ventricular). This report presents a rare case of mid-ventricular TC in a healthy woman in her early thirties, who presented with chest pain in the immediate post-puerperium period following natural delivery. It also outlines how a systematic approach, using the most recent evidence-based guidelines, was followed to reach a final and conclusive diagnosis. Additionally, the report discusses the conceptual hormonal changes that occur in this patient population and their potential role in the development of the condition.

## Case presentation

**Figure 1:**
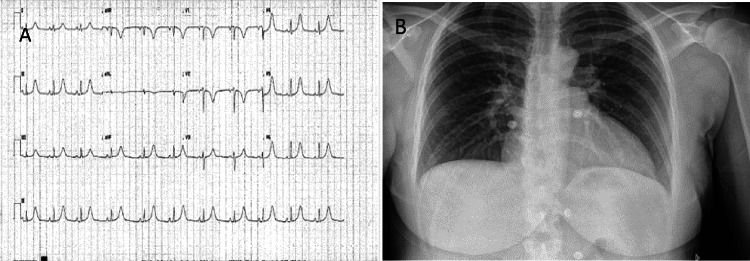
(A) A 12-lead ECG showing hyperacute T-wave and QTc interval prolongation; (B) chest X-ray showing clear lungs. ECG, electrocardiogram

**Figure 2:**
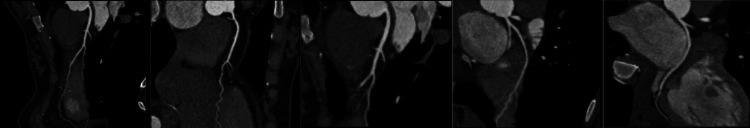
CT coronary angiography showing clear unobstructed coronary arteries.

**Figure 3:**
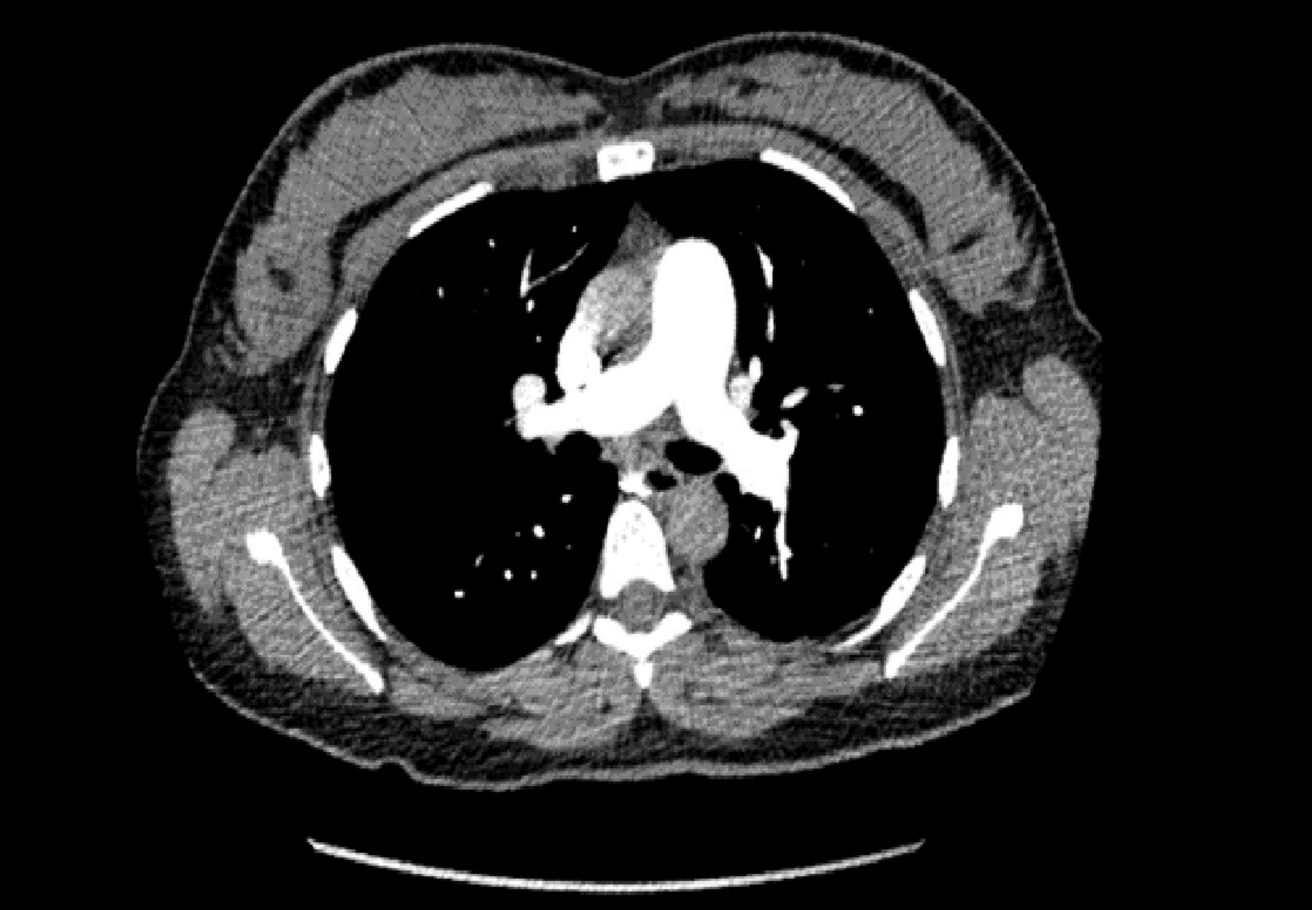
CT pulmonary angiography showing the absence of any evidence of pulmonary embolism.

**Figure 4:**
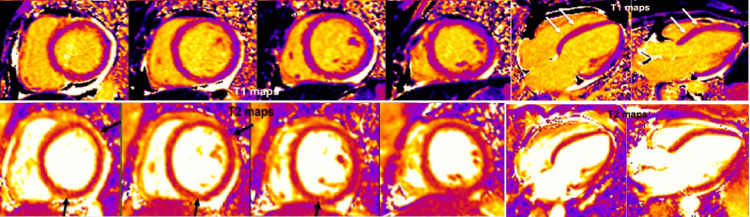
Cardiac magnetic resonance imaging with T1 and T2 mapping. Cardiac magnetic resonance imaging with T1 and T2 mapping shows increased T2 [FISP] mapping values (black arrows) in the basal and mid-LV septal walls, basal-to-mid anterior, basal-to-mid anterolateral, and basal-to-mid inferior walls, with more pronounced changes in mid-LV segments, indicating the presence of edema/inflammation. No LV/RV thrombus was identified in the early phase after gadolinium administration. There is no late gadolinium enhancement. FISP, fast imaging with steady-state precession

A young, healthy 31-year-old ethnically White woman was admitted for an uneventful induced vaginal delivery. Antenatal blood tests showed low pregnancy-associated plasma protein A (PAPPA-A) levels, and she was started on aspirin antenatally to reduce the risk of preeclampsia and growth restriction. Her pregnancy was otherwise uneventful. She developed central, non-radiating pressure-like chest pain two hours after delivery associated with shortness of breath and dizziness. Clinical examination revealed the woman in physical distress but with stable hemodynamic parameters. A chest X-ray was carried out initially, which showed no radiological abnormalities (Figure 1A). Her initial ECG revealed T-wave inversions (TWIs) in leads V1-V3. Subsequent ECGs showed further development of the TWIs and the eventual appearance of new hyperacute T-waves in leads V4-V6, as well as in leads II and aVF, along with progressive prolongation of her QTc (Figure 1B). Laboratory tests showed evidence of myocardial injury with elevation of high sensitivity Troponin I to 1,452 ng/L initially and peaking at 1,673 ng/L the following day. A subsequent discussion with the cardiology team resulted in advice for urgent transfer to the CCU, along with a CT coronary angiogram (CTCA) (Figure 2) and a CT pulmonary angiogram (CTPA) (Figure 3) to rule out coronary artery disease (CAD), spontaneous coronary artery dissection (SCAD), and pulmonary embolism, respectively. Her CTCA showed clear, unobstructed coronary arteries with no evidence of coronary artery dissection. As a result, antiplatelet therapy was not advised, given these findings and the risk of postpartum bleeding. A transthoracic echocardiogram (TTE) performed the following day demonstrated the characteristic pattern of atypical Takotsubo, with severely impaired systolic function of 30%-35% visually and 32.3% by Simpson’s biplane measurements. The study also revealed regional wall motion abnormalities (RWMAs) of a multi-territorial nature, causing akinesia in the mid-basal inferoseptal, anterior, and anteroseptal walls, as well as hypokinesia in the mid-basal anterolateral, inferior, and inferolateral walls. The apical walls and apex, however, maintained preserved function (Video 1). A subsequent cardiac magnetic resonance (CMR) imaging was carried out and confirmed an impaired LVEF of 44%, with akinesis of the basal and mid-LV septal walls, as well as the mid-anterior and mid-inferior walls. More notably, there was evidence of edema/inflammation predominantly confined to the basal to mid-LV segments, with an absence of myocardial fibrosis, scarring, or evidence of previous infarction (Figure 4). These findings confirmed the suspected diagnosis of atypical Takotsubo. To provide further evidence, a repeat TTE performed two days later, before discharge, showed marked improvement in LV function, with an ejection fraction of 50%-55% and only mild hypokinesia in the same segments mentioned earlier (Video 2). Given the marked improvement in her LV function before discharge, the patient was started on a low dose of beta blockers and was later discharged with a plan for a repeat CMR in three months, along with an outpatient cardiology follow-up appointment.

**Video 1:** Transthoracic echocardiogram one day post-presentation. A transthoracic echocardiogram performed one day post-presentation shows the pattern of atypical Takotsubo, with severely impaired systolic function (30%-35% visually) and regional wall motion abnormalities (RWMAs) of a multi-territorial nature. These abnormalities caused akinesia in the mid-basal inferoseptal, anterior, and anteroseptal walls, and hypokinesia in the mid-basal anterolateral, inferior, and inferolateral walls, while the apical walls and apex maintained preserved function.

**Video 2:** Transthoracic echocardiogram done three days after presentation and before discharge. A transthoracic echocardiogram performed just before discharge showed marked improvement in LV function, with values approaching normal. LV function was visually estimated at 50%-55%. LV, lower ventricle

## Discussion

Since it was first described by Sato et al. in 1990, various terms, including apical ballooning syndrome (ABS), stress-induced cardiomyopathy, and broken heart syndrome, have been used to describe conditions with essentially similar characteristics to TC. These conditions present with a pattern of left ventricular dysfunction affecting different segments of the LV wall in response to intense emotional and physiological stressors in the absence of any significant coronary artery disease. The term Takotsubo syndrome has since been used as an umbrella term that covers all of these conditions. While the condition has been shown to affect any age group, it’s most commonly prevalent in women in their sixth decade of life whereas patients under the age of 50 comprise only about <10% of those affected [3]. There are four distinct subtypes of the condition, depending on the pattern of myocardial dysfunction. The most commonly noted pattern of LV dysfunction is characterized by apical hypokinesia and basal hyperkinesia and accounts for 81.7% of cases; other subtypes include the mid-left ventricular type, the focal wall motion type, and the basal wall motion type, which account for 14.6%, 1.5%, and 2.2%, respectively. The basal type is sometimes referred to as reverse or inverted and is mostly seen in younger patients with more neurological disease [3,4,5].

Given the close resemblance between TC and ACS, the international Takotsubo registry created the InterTAK score as a useful clinical predictor for the diagnosis of TC. The score takes factors such as gender, ECG changes, a history of previous psychiatric or neurological disorders, and emotional or physical stressors into account to provide probability predictions that guide management. The confusion between ACS and TC is compounded even more by the fact that not all cases of TC are preceded by triggers. In their paper published in 2016, the international Takotsubo registry identified that physical triggers accounted for 36% of all TC cases, while emotional triggers and a combination of both accounted for 26% and 8% of all cases respectively. Remarkably, no specific identifiable trigger was found in almost one-third of the patients [5].

The relatively fast and marked recovery noted in the LV function post-presentation as compared to ACS drove cardiologists of the past to think of TC as a benign condition and thus historically reassurance, in many cases false, was given to patients after diagnosis. Current literature, however, indicates that patients with TC have an in-hospital mortality rate that is comparable to ST-elevation myocardial infarction and higher rates of all-cause mortality and major adverse cardiovascular and cerebrovascular events even after the acute event when compared to the general population [6].

Despite being first described more than 30 years ago, the etiopathogenic mechanisms of TC remain not fully understood, and the lack of treatment options continues to present a clinical dilemma for most clinicians [2]. The most prevailing theory suggests that myocardial stunning, caused by excessive catecholamine release, drives increased myocardial metabolic demand and coronary vasospasm, leading to a state of perfusion-demand mismatch as the most likely hypothesis [7,8]. Additionally, estrogen deficiency leading to impaired coronary microvascular function is thought to play a role as an underlying mechanism that could explain the higher prevalence of the condition in post-menopausal women [9].

In apical TC, the exact mechanism behind the unique apical ballooning morphonology remains unclear. The beta-receptor gradient theory postulates that an intracellular signaling switch from Gs proteins to Gi proteins in beta-2 adrenergic receptors is induced by the surge in catecholamines, mainly epinephrine. This produces an inhibitory effect on the myocardium. Given that the apex contains the highest concentration of these receptors in the LV, the end result is a stunned apical myocardium with the characteristic apical ballooning morphology. Estrogen through its regulatory effect on B2 receptor gene expression plays an important role in this process and thus possibly explains why this condition is more prominently observed in post-menopausal women and why the abrupt withdrawal of estrogen after pregnancy can explain its triggering effect in post-partum women [10]. In atypical TC, other factors have been suggested to influence epinephrine concentration in the ventricular myocardium, such as the conversion of norepinephrine to epinephrine by phenylethanolamine N-methyltransferase. This enzyme is found in its highest concentration at the point of greatest sympathetic innervation in the basal myocardium, which could explain why myocardial stunning is predominantly basal rather than apical in these cases [10].

The association between pregnancy and TC has long been established. Pregnancy represents a strong physiological stressor for a mother due to the high burden of physiological changes necessary to carry the pregnancy to term [11]. Furthermore, the late stages of pregnancy are characterized by high estrogen levels that are readily exhausted post-partum after placental expulsion. The abrupt withdrawal of estrogen combined with catecholamine administration and beta-agonists used to stimulate uterine contraction precipitates the condition, especially in the immediate puerperium [12]. Interestingly, cesarean delivery is more commonly associated with TC in comparison to natural delivery despite the lower sympathetic activity, which could be explained by a more prominent vagal withdrawal in cesarean deliveries tipping the sympathovagal balance toward the sympathetic side in the former substratum of patients [12].

In our patient’s case, while she underwent a natural vaginal delivery as opposed to a cesarean delivery, it is still recognized as one of the known triggers for TC due to the strong emotional and physical stress associated with labor. Her onset of chest pain, two hours after her delivery, corresponds to the expected time frame for TC to develop in the puerperium. Her ECG showed changes were also consistent with TC in the form of QTc prolongation and hyperacute T-wave changes together with no ST depression. These changes are highly suspicious of TC, especially after a strong physical and emotional trigger such as labor, and are part of the criteria used in the InterTAK score. An InterTAK score of 56 was calculated, which correlates to an approximately 95% predicted probability for TC and a sensitivity of 94.7% [13].

To add more evidence, troponin levels were only moderately elevated, consistent with expected levels in TC, with a peak of 1,673 ng/L in the first 24 hours after the onset of chest pain. This disproportionate level of elevation in contrast with the marked and acute deterioration in LV function with its multi-territorial nature is compatible with TC and consistent with what was noted in previous studies [14]. Khan et al. [15] demonstrated the value of the brain natriuretic peptide (BNP)/Troponin I ratio in differentiating between TC and MI patients. TC patients were shown to have ratios ranging from 331.8 to 1,226.5 pg/µg, in comparison to MI patients, who had ratios between 50.5 and 372.3 pg/µg.

As per the recommendations of the Heart Failure Association Takotsubo Task Force and the International Takotsubo Registry criteria [8], coronary artery disease and dissection must be ruled out before a diagnosis of TC can be made. Coronary angiography is the modality of choice for coronary assessment; however, expert consensus advises that CTCA can be used in cases where angiography poses a high risk, especially when there is a high suspicion of TC [16]. In our patient's case, CTCA was preferred over coronary angiography due to the risk of postpartum bleeding. The results confirmed the absence of CAD or obstruction, further strengthening the argument for a TC diagnosis.

CMR is uniquely suited for TC patients, as it not only allows for accurate estimation of RWMAs and both left and right ventricular function, but more importantly, it provides a valuable marker for reversible conditions like myocardial edema and inflammation, as opposed to more irreversible conditions such as fibrosis or scarring. In our case, CMR provided the final piece for a conclusive diagnosis. T2 mapping revealed areas of edema and inflammation, more pronounced in the mid-LV segments, along with the RWMAs noted above. More importantly, late gadolinium enhancement (LGE) was absent. Eitel et al. [17] demonstrated that while the exact pathophysiology behind myocardial edema in TC patients is not fully clear, inflammation combined with increased LV stress and possible transient ischemia plays a major role in the development of the condition as the high pressure inside the LV could catalyze or even provoke focal perfusion defects. The team also demonstrated that, while the absence of LGE has been previously described as a diagnostic criterion, their study showed that about 9% of cases had minute or patchy myocardial scarring. Those with evidence of even minor LGE seemed to have significantly higher troponin levels at presentation.

Takotsubo syndrome lacks randomized clinical trials for specific treatment guidelines. In-hospital care should focus on risk stratification and supportive measures, with mild cases requiring little to no treatment and severe cases potentially needing mechanical support. Heart failure medications, including beta-blockers such as metoprolol and carvedilol, should be considered for patients with an LVEF of 35-40%, provided there are no contraindications. Other opinions suggest avoiding vasoactive drugs, including ACE inhibitors, in patients with normal LVEF, as low peripheral resistance has been implicated in altered peripheral sympathetic nerve activity in TC patients [4]. In our patient's case, the marked improvement in her LV function within just three days was reassuring from a clinical perspective. As a result, she was started on beta-blockers at discharge, with a plan for follow-up in three months and repeat CMR before the appointment.

## Conclusions

TC remains one of the leading confounders for ACS worldwide. In this report, we demonstrate how a guideline-based approach helped us readily identify and manage a rare case of atypical Takotsubo in an otherwise healthy woman following a natural vaginal delivery, utilizing multiple investigative modalities, specifically transthoracic echocardiography and CMR. We also highlighted how the InterTAK score was crucial in guiding clinical suspicion and decision-making. Future studies are needed to investigate the role of postpartum hormonal changes in the development of cardiac dysfunction in this patient population, as well as to devise optimized therapeutic options.
